# Significance of hydrogen breath tests in children with suspected carbohydrate malabsorption

**DOI:** 10.1186/1471-2431-14-59

**Published:** 2014-02-27

**Authors:** Jan Däbritz, Michael Mühlbauer, Dirk Domagk, Nicole Voos, Geraldine Henneböhl, Maria L Siemer, Dirk Foell

**Affiliations:** 1 Department of Pediatric Rheumatology and Immunology, University Children’s Hospital Münster, Röntgenstr 21, Münster 48149, NRW, Germany; 2 The Royal Children’s Hospital Melbourne, Murdoch Children’s Research Institute, Gastrointestinal Research in Inflammation & Pathology, Parkville 3052, VIC, Australia; 3 Melbourne Medical School, Department of Pediatrics, University of Melbourne, Parkville 3052 VIC, Australia; 4 Pediatric Surgery, Mathias Hospital Rheine, Rheine 48431, NRW, Germany; 5 Department of Gastroenterology (Medicine B), University Hospital Münster, Münster 48149, NRW, Germany; 6 Center for Clinical Trials, University Hospital Münster, Münster 48149, NRW, Germany

**Keywords:** Carbohydrate intolerance, Gastrointestinal disease, Fructose, Lactose, Sorbitol

## Abstract

**Background:**

Hydrogen breath tests are noninvasive procedures frequently applied in the diagnostic workup of functional gastrointestinal disorders. Here, we review hydrogen breath test results and the occurrence of lactose, fructose and sorbitol malabsorption in pediatric patients; and determine the significance of the findings and the outcome of patients with carbohydrate malabsorption.

**Methods:**

We included 206 children (88 male, 118 female, median age 10.7 years, range 3–18 years) with a total of 449 hydrogen breath tests (lactose, n = 161; fructose, n = 142; sorbitol, n = 146) into a retrospective analysis. Apart from test results, we documented symptoms, the therapeutic consequences of the test, the outcome and the overall satisfaction of the patients and families.

**Results:**

In total, 204 (46%) of all breath tests were positive. Long-term follow-up data could be collected from 118 patients. Of 79 patients (67%) who were put on a diet reduced in lactose, fructose and/or sorbitol, the majority (92%, n = 73) reported the diet to be strict and only 13% (n = 10) had no response to diet. Most families (96%, n = 113) were satisfied by the test and the therapy. There were only 21 tests (5%) with a borderline result because the criteria for a positive result were only partially met.

**Conclusions:**

Hydrogen breath tests can be helpful in the evaluation of children with gastrointestinal symptoms including functional intestinal disorders. If applied for a variety of carbohydrates but only where indicated, around two-third of all children have positive results. The therapeutic consequences are successfully relieving symptoms in the vast majority of patients.

## Background

Hydrogen breath tests (HBT) are noninvasive investigations frequently used in pediatric gastroenterology. The range of diseases that can be identified include carbohydrate intolerance, small intestinal bacterial overgrowth, and orocecal transit time. In the normal individual, gut bacteria are primarily located in the colon and the distal small intestine. When defective sugar absorption is present, unabsorbed carbohydrates make up the substrate for the saccharolitic flora of the colon. Short-chain fatty acids and gases (e.g. hydrogen [H_2_], methane [CH_4_]) are formed from the metabolism of this flora. Human cells do not produce H_2_ and CH_4_. The rationale of HBT is based on the concept that part of the gas produced by colonic bacterial fermentation diffuses into the blood and is excreted by breath, where it can be quantified easily by chromatography [1].

Lactose maldigestion occurs when lactose is not hydrolyzed in the small intestine. It passes into the colon and through increased fluid secretion and gas production can lead to symptoms of lactose intolerance including abdominal pain, diarrhea, flatulence, bloating, nausea or vomiting. In some cases subjects can present with constipation and others are detected only because their weight gain is inadequate. A diagnosis of lactose intolerance in children requires exclusion of other clinical diagnoses such as allergy to cow's milk proteins or other foods, or intolerance to other disaccharidases [1]. The HBT with lactose is still considered to be the most reliable, noninvasive and economical technique to measure lactose maldigestion.

Fructose is a monosaccharide occurring naturally in its free form or as sucrose (glucose plus fructose) and it is absorbed by carrier-mediated facilitated diffusion [2]. Fructose is widely used as a sweetener in foods, beverages, and candy, and it is also present in a variety of fruits. Ingestion of high levels of fructose can lead to carbohydrate intolerance and may results in nonspecific diarrhea, excessive intestinal gas, and recurrent abdominal pain. Sorbitol is a sugar alcohol widespread in plants, particularly in fruits and juices. Sorbitol is normally only partially absorbed and simultaneous ingestion of sorbitol and fructose seems to increase malabsorption of the latter [3,4]. Methodology and interpretation of results are similar to HBT with lactose. However, the role of HBT with fructose and sorbitol is uncertain and to date often not recommended in clinical practice [5].

HBT have a special role in the diagnostic workup of functional gastrointestinal disorders in children. However, the exact read out (e.g. rise of hydrogen values over baseline) and the significance of the findings (with many false-positive results) especially in children remain a matter of debate [6]. There is a need for rationalizing the use of HBT tests in order to standardize test procedures and optimize their use in pediatric clinical practice. Here we review hydrogen breath test results and the occurrence of carbohydrate malabsorption in pediatric patients and evaluate the significance of the findings, and the outcome of patients.

## Methods

### Patients

In our retrospective single-centre cross-sectional study we analysed a pediatric cohort of patients who underwent extended-panel breath testing for diagnostic workup of unspecific chronic abdominal symptoms. We included children and adolescents with symptoms of functional bowel disorders or chronic/recurrent abdominal pain syndrome based on currently accepted Rome III criteria for these diagnoses [7,8]. Pediatric patients were referred to the gastroenterology breath-testing clinic at the University Hospital Münster between January 2005 and August 2010 by pediatricians and/or pediatric gastroenterologists for testing of carbohydrate malabsorption. Some patients were referred for testing with a single carbohydrate, and many completed a panel of carbohydrate malabsorption tests on different days, including fructose, lactose, and sorbitol.

The indication of breath tests was approved by experienced board-certified pediatric gastroenterologists at the Dept. of Pediatric Gastroenterology of the University Children’s Hospital Münster (Germany). Chronic/recurrent abdominal pain was defined by pain of at least three months' duration. Clinical evaluation of included patients was based on history, physical examination, pain diary, and stool testing for occult blood to identify potential indications of an organic etiology. Additional investigations (including other laboratory tests, radiologic evaluation and/or endoscopy) were performed in the presence of ‘alarm findings’ (e.g. fever, gastrointestinal bleeding, weight loss, anemia, abdominal mass etc.) [9]. Our stepwise diagnostic approach was in accordance with current clinical guidelines [10] and allowed exclusion of other underlying gastrointestinal diseases in our study cohort.

All included patients with positive HBT results received dietary counselling by the responsible pediatrician at the Dept. of Pediatric Gastroenterology of the University Children’s Hospital Münster. In addition, all included patients with positive test results were offered further dietary counselling by an experienced pediatric dietician at the University Children’s Hospital Münster. Patients and/or parents were asked to start the restriction-diet by avoiding or cutting down on foods that contain lactose, fructose and/or sorbitol, respectively. In cases with multiple sugar malabsorption diet was restricted to all sugars that were tested positive. Patients and/or parents were provided with written patient information including specific health information fact sheets and tables on lactose-, fructose- and/or sorbitol-containing food products. Patients and/or parents were instructed to read labels of commercially prepared foods. Information on effective label reading was provided. It was ensured that protein, fat, and other nutrients were supplied at appropriate levels.

Patient’s parents/caretakers were asked to fill out a follow-up questionnaire at a review visit 15 month after the initial breath test(s) to determine i) abdominal symptoms (pain/diarrhea/other/unknown) before and during the HBT; ii) HBT results (normal/abnormal/unknown); iii) diet counseling attended (yes/no), and duration of diet (<6/6-12/>12 months); iv) adherence to dietary restrictions (yes/no), degree of adherence to dietary restrictions (always/mostly/rarely/never) and success of dietary restrictions (yes/partly/no); v) initiation of further investigations following a positive HBT (yes/no); vi) overall satisfaction with HBT (yes/no) and diet counseling (yes/no). Questionnaire answers regarding i) abdominal symptoms before and during the HBT, ii) HBT results, iii) further investigations, and iv) diet counseling attendance were confirmed by the authors based on clinical records. The study was approved by the institutional review board and informed consent was obtained from all patients and/or parents for the inclusion into the retrospective analysis.

### Pretest conditions

Patients were asked to fast from solids and liquids for a minimum of 8 hours (overnight) before the test and to have only small amounts of water; tests were conducted in the morning and patients fasted from midnight. In order to avoid false-positive and false-negative results, patients were asked to eat a low-fibre evening meal, not to have exercised in the morning of or during the test, and not to have taken antibiotics, probiotics, prokinetics, laxatives and/or solutions for colonic clearing within 4 weeks before the test. Good oral hygiene was also recommended. Patients were excluded for bowel resection, malignancies, small bowel obstructions, pancreatic divisum, malabsorption syndrome, pancreatic insufficiency, or other comorbid illnesses. Patients with an already established diagnosis of *Helicobacter pylori* infection, inflammatory bowel disease, coeliac disease, parasite infections, or small bowel bacterial overgrowth were also excluded.

### Hydrogen breath test

Patients received lactose (AppliChem, Darmstadt, Germany) at a dose of 1 g/kg body weight (20% water solution; maximum dose 50 g), fructose (AppliChem, Darmstadt, Germany) at a dose of 0.5 g/kg body weight (10% water solution; maximum dose 25 g), or sorbitol (AppliChem, Darmstadt, Germany) at a dose of 0.2 g/kg body weight (10% water solution; maximum dose 10 g) in a drink of 6 ml/kg (maximum volume 300 ml) [5,11-14]. Patients could have more than one test, but only at different days. Breath samples were collected at 15-minute intervals after sugar ingestion, for a total of up to 180 minutes. Breath samples were collected by a maximal inspiration followed by a short period of apnea (15 seconds, if possible) and a prolonged expiration through an efficient device [5]. Hydrogen levels of end-expiratory breath samples were analyzed immediately after collections on a stationary GMI H_2_-Analyzer (Stimotron, Wettenberg, Germany), and recorded by a trained technician. Baseline values for hydrogen (after overnight fasting) were measured. Breath tests were interpreted by an experienced gastroenterologist, who was blinded to subject conditions and symptoms. An increase in the production of hydrogen ≥20 parts per million (ppm) and a two-fold increase of the individual baseline H_2_-exhalation (if basal level was ≥10 ppm) in three consecutive samples was considered positive [5,11], but post-hoc adaptation of the threshold was a pre-specified secondary aim. During the test, the patients reported any symptoms. If the patient was unable to tolerate the procedure because of severe abdominal pain, nausea, vomiting, gas, or flatus, the test was terminated early.

### Data analysis

For continuous variables, median and range were documented except when otherwise stated. For categorical variables, percentages are provided. Hydrogen concentrations are expressed as mean ± standard error of the mean (SEM). Graphs were produced in GraphPad Prism Version 5.00 for Windows (GraphPad Software, La Jolla, California, USA).

## Results

### Patients and overall test results

In total, 206 white pediatric patients (Caucasian descent) were included into the study with a total of 449 hydrogen breath tests. Clinical and demographic characteristics of the study subjects are summarized in Table 1. In 60 patients, only a single test was performed, 49 received tests for two carbohydrates, while 97 had tests for three sugars. In total, 204 (45%) of all tests were positive (Table 1). In 63 children (31%), none of the tests were positive. Many of the symptoms previously reported by the patients were reproduced by the ingestion of carbohydrates and were experienced during the hydrogen breath test and included abdominal pain, diarrhea, skin blushing, or nausea (Table 1). Patients with negative breath test results also reported symptoms during the test. In few patients (2%), the test needed to be interrupted before reaching a definite result based on breath hydrogen because of severe complaints such as pain, vomiting, or diarrhea (fructose, n = 4; lactose, n = 1, sorbitol, n = 3). Detailed information on initial symptoms (abdominal pain, diarrhea, or both) was not available for all included patients. However, all included patients received HBTs on the background of symptoms of chronic/recurrent abdominal pain. Detailed information and follow-up data were available for 57% of the included patients (n = 118) and given in Table 1.

**Table 1:** Characteristics of patients and test results

	**All**	**3-5 yrs**	**6-8 yrs**	**9-11 yrs**	**12-14 yrs**	**15-18 yrs**	**Male**	**Female**	**Follow-up patients**
**Patients**, n (%)	206 (100)	23 (11)	47 (23)	51 (25)	32 (16)	51 (25)	87 (42)	119 (58)	118 (57)
**Sex**, female/male ratio	1.3	0.9	1.5	1.0	1.8	1.7	—	—	1.4
**Age**, years (median)	10.7	4.3	7.2	10.0	12.9	16.3	10.3	11.1	10.5
**Indication**, n (%)									
Abdominal pain	N/A	—	—	—	—	—	—	—	66 (56)
Diarrhea									6 (5)
Abdominal pain + diarrhea									36 (31)
Other									10 (8)
**Breath tests**, n (% positive)									
Total	449 (45)	44 (41)	107 (45)	117 (44)	73 (52)	108 (41)	190 (38)	259 (49)	261 (46)
Fructose	142 (39)	6 (46)	19 (56)	15 (38)	10 (42)	5 (16)	21 (36)	34 (41)	87 (39)
Lactose	161 (22)	4 (19)	6 (14)	9 (24)	6 (26)	11 (29)	16 (22)	20 (23)	90 (23)
Sorbitol	146 (75)	8 (80)	23 (74)	28 (68)	22 (85)	28 (74)	36 (62)	73 (83)	84 (79)
**Symptoms at test performance**, n (%)									
Abdominal pain	N/A	—	—	—	—	—	—	—	68 (58)
Diarrhea									11 (9)
Abdominal pain + diarrhea									44 (37)
Skin blushing									10 (8)
Nausea									8 (7)
Constipation									2 (2)
Other									8 (7)
**Multiple tests**, n (%)									
1 test performed	60 (29)	10 (43)	14 (29)	11 (20)	7 (19)	18 (38)	29 (33)	31 (26)	35 (30)
2 tests performed	49 (24)	5 (22)	9 (19)	13 (26)	8 (26)	11 (21)	13 (20)	33 (28)	25 (21)
3 tests performed	97 (47)	8 (35)	25 (52)	27 (54)	17 (55)	22 (41)	45 (52)	54 (46)	58 (49)
**Multiple test results**, n (%)									
All negative	63 (31)	10 (43)	16 (34)	15 (29)	7 (22)	15 (29)	36 (41)	27 (23)	33 (28)
1 of 3 tests positive	38 (18)	3 (13)	9 (19)	8 (16)	8 (25)	10 (20)	13 (15)	25 (21)	24 (20)
2 of 3 tests positive	37 (18)	1 (4)	10 (21)	13 (25)	7 (22)	6 (12)	18 (21)	19 (16)	21 (18)
1 of 2 tests positive	30 (15)	3 (13)	7 (15)	6 (12)	3 (9)	10 (20)	9 (10)	21 (18)	11 (9)
1 of 1 test positive	21 (10)	4 (17)	2 (4)	6 (12)	2 (6)	6 (12)	7 (8)	13 (11)	8 (8)
2 of 2 tests positive	10 (5)	1 (4)	2 (4)	2 (4)	4 (13)	1 (2)	1 (1)	9 (8)	16 (14)
3 of 3 tests positive	7 (3)	1 (4)	2 (4)	1 (2)	1 (3)	2 (4)	3 (3)	4 (3)	5 (4)

### Test performances at different cut-offs

A definitive positive test results with an increase in the production of hydrogen ≥20 ppm plus a two-fold increase of the individual baseline H_2_-exhalation in three breath samples was observed in 192 breath tests (fructose, n = 59; lactose, n = 31; sorbitol, n = 102). There were only 21 tests (5%) with a borderline result because the criteria for a positive result were only partially met. Reasons were: i) three hydrogen values ≥20 ppm but not all of those topped the baseline value by a two-fold increase (fructose, n = 1; lactose, n = 3; sorbitol, n = 3); or ii) hydrogen values reached ≥20 ppm, but not at three time points (fructose, n = 7; lactose, n = 3; sorbitol, n = 4). A baseline hydrogen value ≥10 ppm was noticed in 61 breath tests (fructose, n = 19; lactose, n = 26; sorbitol, n = 16) and test results were positive in 51% of these cases (fructose, n = 9; lactose, n = 9; sorbitol, n = 13). In total, 36 HBTs (8%) showed elevated baseline hydrogen levels (>20 ppm) at 15–30 min after sugar ingestion (fructose, n = 20; lactose, n = 9, sorbitol, n = 7). In 118 patients, both fructose and sorbitol tests were performed. In 23 of these patients (20%), both tests were negative, and 39 patients (33%) had positive results in both tests. In the 56 patients (47%) with only one out of two positive tests, only 5 patients had an abnormal fructose breath test while 51 were diagnosed with sorbitol-intolerance. Testing for sorbitol may thus be meaningful, both in combination and independently.

**Figure 1:**
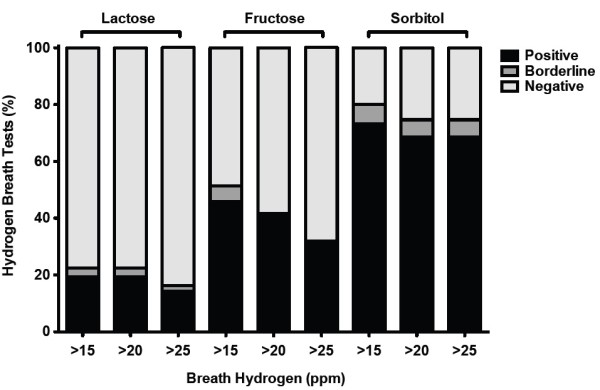
**Performance of the hydrogen breath tests at different hydrogen cut-offs.** Percentage of positive, negative and borderline hydrogen breath test results by three different hydrogen breath cut-offs as indicated. An increase in the production of hydrogen ≥20 (≥15; ≥25) ppm and a two-fold increase of the individual baseline H_2_-exhalation in three consecutive breath samples was considered positive. Breath test, in which three hydrogen values were ≥20 (≥15; ≥25) ppm but not all of those topped the baseline value by a two-fold increase or where hydrogen levels reached at least 20 (≥15; ≥25) ppm but not at three time points were considered as borderline. All other test results were classified as being negative.

As expected, higher cut-off levels (i.e. 25 ppm) decreased and lower cut-off levels (i.e. 15 ppm) increased the proportion of positive test results, especially for fructose HBT. However, with regard to the overall test performance and avoidance of borderline results, the cut-off definition at 20 ppm appears reasonable. The performance of different hydrogen breath tests at different cut-offs is shown in Figure 1.

**Figure 2:**
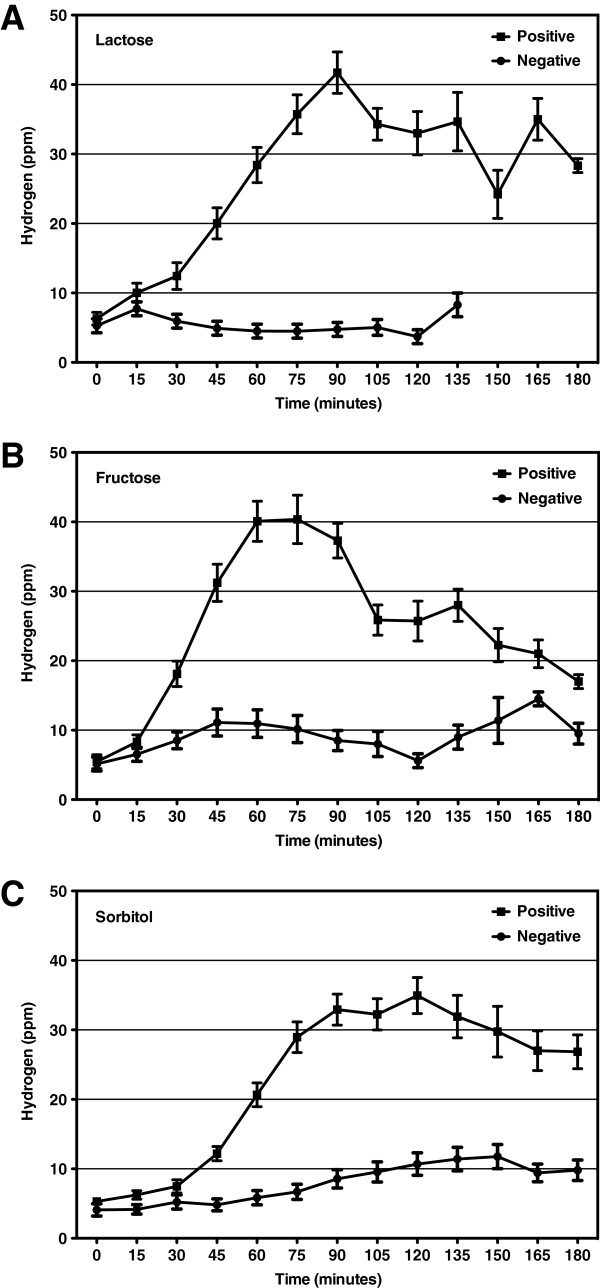
**Time course analysis of hydrogen concentrations during breath tests.** Comparison of the rise in breath hydrogen over time after ingestion of test meal in patients with positive and negative results of hydrogen breath tests with lactose **(A)**, fructose **(B)**, and sorbitol **(C)**. Tracing of breath hydrogen excretion is expressed as mean ± SEM.

We analyzed hydrogen values of tests in which the ingested carbohydrate was absorbed without colonic fermentation and where no hydrogen was produced (negative test). Likewise, breath hydrogen concentrations obtained from patients who were unable to absorb all of the ingested carbohydrate were analyzed at baseline and at different time points after ingestion of the test meal (positive test). In these patients, bacterial fermentation resulted in the appearance of breath hydrogen that was below 20 ppm for the first 45 minutes (lactose), 30 minutes (fructose), or 60 minutes (sorbitol) and reached a maximum value by 90 minutes (lactose), 75 minutes (fructose), or 120 minutes (sorbitol). The maximum hydrogen produced represents peak carbohydrate fermentation. The time course analyses of breath hydrogen concentrations during breath tests using lactose, fructose, or sorbitol are summarized in Figure 2.

### Therapeutic consequences

Long-term follow-up data could be collected from 118 patients (Table 2). Seventy-seven patients (65%) had been reported abnormal findings and 58 patients (75%) had further dietary counseling by a pediatric dietician. Seventy-nine patients (103%) were put on a diet reduced in lactose, fructose and/or sorbitol (in 55 patients [47%] for longer than 12 months). Two patients were put on diet by their parents even though the HBT result was normal. The majority (n = 73; 92%) reported the diet to be strict and only 10 patients (13%) had no response to diet. Most families (n = 113; 96%) were satisfied by the hydrogen breath test and the therapy. In patients with only one positive test, carbohydrate-reduced diet was introduced in 45 cases (38 with success, 5 without, 2 unknown). In patients with 2 positive tests, a diet was introduced in 27 cases (22 with success, 5 without). In patients with 3 positive tests, a diet was introduced in 5 cases (all with success). Follow up data was available for 31 patients with positive sorbitol HBT but negative fructose HBT. Of these patients, 87% (n = 27) reported a benefit of dietary sorbitol-restriction (no sorbitol-containing candies, chewing gums and diet foods), whereas only 13% (n = 4) stated that the sorbitol-restricted diet had no effect on abdominal symptoms.

**Table 2:** Therapeutic consequences

	**With follow-up data**
**Patients**, n	118
**With positive test result**, n (%)	77 (65)
**With nutrition advice**, n (%)	58 (75)
Patients put on diet by parents*, n (%)	79 (103)
**Duration of diet**	
<6 months, n (%)	35 (30)
6-12 months, n (%)	17 (14)
>12 months, n (%)	55 (47)
not specified, n (%)	11 (9)
Patients on strict diet**, n (%)	73 (92)
**Success of dietary measures**	
Complete success, n (%)	47 (59)
Partial success, n (%)	22 (28)
Unsuccessful, n (%)	10 (13)
**Patients/Parents happy with test**, n (%)	113 (96)

*diet reduced in fructose, lactose, sorbitol or a combination of these sugars.

**according to parents.

## Discussion

This was a large retrospective analysis, which allowed us to examine the use of HBT in children and adolescents presenting with chronic/recurrent abdominal symptoms. The aim of our study was to describe the occurrence of carbohydrate malabsorption and the role of HBT for the diagnosis and outcome of these patients. Due to the retrospective design of our study we did not aim to provide data on HBT standardization and validation. In pediatric field there is no agreement on the methodological aspects of HBT even though the modifications of the test meal, the substrate dose, the test performance and the data analysis can have a marked influence on the test results. Studies in children have included lactose doses ranging from 0.5 to 2.0 g/kg, up to an absolute/maximum dose of 25–50 g [1,5,14]. No gold standard is available for the diagnosis of other sugar malabsorption and fructose as well as sorbitol HBT are therefore not recommended in clinical practice [5]. However, a dose of 25 to 50 g of fructose and 5 to 10 g of sorbitol were recommended for these HBTs [12]. Recent studies in children on HBT with fructose used a dose of 0.5 to 1 g/kg to a maximum of 10 to 50 g [15-17]. Thus, the clinical importance of sorbitol and fructose malabsorption and standardized test protocols remains to be elucidated.

We found that HBT in children with nonspecific persistent/recurrent abdominal symptoms were positive in 39% of tests with lactose, 22% of tests with fructose, and 75% of tests with sorbitol. The study by Gomara *et al*. determined the occurrence of fructose malabsorption in pediatric patients with previous diagnoses of a functional bowel disorder and reported similar results with positive tests in 34% of the cases and rapid improvement of gastrointestinal symptoms with dietary fructose restriction [16]. An even higher number of positive HBT with fructose in children (62%) was determined in the study by Tsampalieros *et al*. [17]. Jones *et al*. carried out a retrospective analysis on HBT of symptomatic patients (≤15 years old) and 58% and 40% of these children were tested positive on the fructose and lactose HBT, respectively. Importantly, this study showed a progressive decline in the percentages of positive fructose HBT with increasing age, whereas age did not have a significant effect on the test result for the lactose HBT [15]. In our study, age also had a significant effect on fructose malabsorption, with a higher proportion of young children testing positive, and no effect on the test results for the lactose and sorbitol HBT (Table 1). Our data were also consistent with the limited ability to absorb sorbitol in humans [5,18]. Interestingly, sorbitol HBT results were more often positive in our female patients compared with male patients, whereas patients’ sex had no effect on the test results for the fructose and lactose HBT (Table 1). The high proportion of positive sorbitol HBT in symptomatic children raises the question of the diagnostic value of the test. However, in patients presenting with gastrointestinal symptoms, the HBT will be of value in assessing whether patients have fructose and/or sorbitol malabsorption because at least 40% of our patients had a positive sorbitol HBT but a negative fructose HBT. It has been shown previously that sorbitol, when ingested with fructose has at least additive and possibly synergistic effects in terms of breath hydrogen production and symptom generation. Gibson *et al*. underlined that - despite the absence of quantitative population data and studies investigating whether there are low and high fructose absorbers - most current data suggests that fructose malabsorption is not to be considered abnormal. However, the reaction and sensitivity of the bowel to the altered intestinal environment seems to be factor distinguishing symptomatic and asymptomatic patients [18]. Our results of multiple testing for fructose and sorbitol suggest that sorbitol HBT is in general meaningful. This seems relevant, given the role of sorbitol (in addition to lactose and fructose) ingestion with contemporary dietary habits especially of children in industrialized countries. Furthermore, sorbitol HBT may be a useful screening tool to detect coeliac patients [19]. However, we did not systematically screen patients in our study for coeliac disease and patients with an already established diagnosis of coeliac disease were excluded from our study. Future studies should therefore further address the question whether sorbitol HBT may be a useful screening tool to detect coeliac patients.

HBT results may not always correlate with the production of gastrointestinal symptoms. Studies in adults and children have shown individuals with negative breath test results reported (mainly abdominal) symptoms during the test [16,20]. These symptoms may be due to a hyperosmolar effect, assuming incomplete small intestinal absorption of the respective carbohydrate. If unabsorbed, excessive carbohydrates could serve as an osmotic load that draws fluid into the intestinal lumen, generating gastrointestinal symptoms. It is also possible that the occurrence of symptoms during a negative HBT may in some instances result from the effect of an inactive substance (or procedure) administered suggesting that it will negatively modify a symptom or a sensation ("nocebo effect"). Data emerging from a recent study demonstrate the role of the "nocebo effect” in inducing abdominal symptoms in subjects with a negative carbohydrate HBT [21]. Skin blushing (sudden reddening of the face, neck, or upper chest) during the HBT was reported in 10 of our 118 patients with follow-up data. Skin blushing is a normal body response that may occur when someone is excited or experiencing some other emotion. Therefore, skin blushing may, like other symptoms associated with carbohydrate ingestion, not necessarily be related to sugar malabsorption [22]. In addition, some of the children in the group whose breath test results were negative may have had symptoms during the test related to an underlying functional bowel disorder, not as a result of the ingestion or malabsorption of carbohydrates. Increased sensation in the gastrointestinal tract in response to stimuli of various receptors in the gut wall (visceral hypersensitivity) is a frequent finding in IBS patients and triggered by bowel distention or bloating. Sugar malabsorption might cause problems in patients with visceral hypersensitivity while it still remains tolerated in healthy individuals. In such cases a restricted diet might decrease bowel distention and bloating and therefore improve IBS symptoms. In this regard it has been shown that a fructose-reduced diet improves symptom scores in IBS patients independent of results from the fructose HBT, which indicates that dietary fructose may contribute to the complaints in IBS, and therefore fructose-reduced diet can have therapeutic implications [23,24]. Application of a fructose-restricted diet to patients with IBS and fructose malabsorption has revealed a high level of sustained adherence associated with a high rate of symptomatic improvement [25] and balancing dietary fructose and glucose may mitigate clinical symptoms for those individuals with apparent sensitivity to fructose [26].

It has been shown that malabsorption of lactose, fructose and/or sorbitol has a similar frequency in patients with IBS than in healthy subjects [27,28]. Importantly, it has also been shown that in IBS patients with small bowel bacterial overgrowth (SBBO), sugar breath tests may be falsely abnormal [29]. An abnormal lactulose HBT was found in children with functional gastrointestinal symptoms and additional investigation has been suggested to rule out the underlying cause (e.g. accelerated intestinal transit or SBBO) [30]. However, we did not systematically screen included patients for SBBO and we excluded patients with an already established diagnosis of SBBO. Furthermore, included patients underwent HBT on the background of chronic/recurrent abdominal pain and included patients did not have an established diagnosis of IBS. It would be worthwhile to investigate the link between IBS, SBBO and HBT in future studies in more detail. However, this was beyond the scope of our present study. It will also be important in future studies with a blinded and controlled design to determine how symptoms should be assessed during the test and to what extent these are reliable [22]. Further studies are also needed to address research goals with regard to fructose intolerance, standardisation of symptom assessment during the test and long-term follow up, and sequential reintroduction of sugars in multiple sugar malabsorption. Furthermore, new analytical methods with a semi-quantitative interpretation of breath tests might provide a broader understanding of breath test outcomes [31]. Nevertheless, we imposed dietary restrictions only in patients with positive breath test results despite the finding that patients with negative breath test results reported symptoms after carbohydrate ingestion. We did this because of the unreliability of symptom reporting previously shown during carbohydrate malabsorption studies and the high reliability of hydrogen breath testing to detect incomplete carbohydrate digestion [32,33].

A small percentage of patients with a negative breath test result may have not had an adequate amount of hydrogen-producing bacteria resulting in a negative breath test result. Hydrogen nonproducers harbor a colonic flora unable to produce hydrogen during fermentation and the reported prevalence varies widely from 2 to 43% [5]. Breath methane excretion represents an alternative gaseous marker for intestinal gas breath excretion measurement in this subgroup of subjects, thus possibly improving the test accuracy [5,34]. Furthermore, high baseline hydrogen values may indicate SBBO but this finding is very unspecific as this may also be due to slow intestinal transit with unabsorbable carbohydrates still in the colon. We did not perform further specific testing for SBBO (e.g. glucose HBT). Most observed elevated baseline values are usually explained by an inappropriate fasting period and/or high fibre diet before the test [12].

We demonstrated that significant lactose, fructose and/or sorbitol restriction resulted in immediate relief of symptoms in the majority of patients with positive breath test results. Although compliance with a carbohydrate-restricted diet may be difficult, our patients likely maintained their diet restriction at 15 month of follow-up because of improvement in their symptoms. Previous studies and our clinical experience support the benefit of evaluation for carbohydrate malabsorption using HBT in patients with unexplained chronic abdominal pain [2,16,20,35]. Our findings also support the recommendation of lactose, fructose and/or sorbitol restriction as an easily implemented therapy in children that can result in a decrease in gastrointestinal symptoms.

## Conclusions

Although the retrospective nature of our analyses is a clear limitation, our results support hydrogen breath tests as noninvasive and sensitive measures of carbohydrate malabsorption that can be helpful in the evaluation of children with gastrointestinal symptoms including functional intestinal disorders. A thorough history taking can guide the evaluation of single or multiple sugars to be tested. If applied for a variety of carbohydrates but only where indicated, around two-third of all children have positive results. The therapeutic consequences are successfully relieving symptoms in the vast majority of patients. The adherence to the physician’s advice is excellent, as the patients and families are very content.

## Competing interests

The authors declare that they have no competing interests.

## Authors’ contributions

DF, MM, DD and JD planned the study and led the clinical trial. MM, JD and DF collected and analyzed the data, interpreted the results and did the statistical analyses. DD and MLS performed and validated hydrogen breath tests. NV and GH helped with collecting follow-up data. JD and DF wrote the manuscript. Each author has approved the final version of the report and takes full responsibility for the manuscript. JD and MM contributed equally to this work.

## Pre-publication history

The pre-publication history for this paper can be accessed here:

http://www.biomedcentral.com/1471-2431/14/59/prepub
